# Monument to Dr. Jenner

**Published:** 1852-01

**Authors:** 


### MONUMENT TO DR. JENNER.

We are gratified to learn that the subscription to this testimonial of the world's appreciation of Jenner's invaluable discovery, is progressing favorably in this city. We stated in a previous number that the originators in the movement were desirous that the opportunity of contributing towards it should not be confined to England, and that local committees would be appointed in the principal cities. In this city Drs. Robley Dunglison, G. B. Wood, and T. D. Mütter, have consented to serve, by whom we are authorized to say, that the smallest as well as the most liberal donations will be received.

